# A general modeling framework for exploring the impact of individual concern and personal protection on vector-borne disease dynamics

**DOI:** 10.1186/s13071-022-05481-7

**Published:** 2022-10-08

**Authors:** Kimberlyn Roosa, Nina H. Fefferman

**Affiliations:** 1 One Health Initiative, University of Tennessee, Knoxville, TN USA; 2 National Institute for Mathematical and Biological Synthesis (NIMBioS), University of Tennessee, Knoxville, TN USA; 3 Department of Ecology & Evolutionary Biology, University of Tennessee, Knoxville, TN USA

**Keywords:** Vector-borne disease, Personal protection, *Aedes aegypti*, Mosquito-borne, Dynamic model, Compartmental model, Zika, Theoretical model, Computational simulation

## Abstract

**Background:**

As climate variability and extreme weather events associated with climate change become more prevalent, public health authorities can expect to face an expanding spectrum of vector-borne diseases with increasing incidence and geographical spread. Common interventions include the use of larvicides and adulticides, as well as targeted communications to increase public awareness regarding the need for personal protective measures, such as mosquito repellant, protective clothing, and mosquito nets. Here, we propose a simplified compartmental model of mosquito-borne disease dynamics that incorporates the use of personal protection against mosquito bites influenced by two key individual-level behavioral drivers—concern for being bitten by mosquitos as a nuisance and concern for mosquito-borne disease transmission.

**Methods:**

We propose a modified compartmental model that describes the dynamics of vector-borne disease spread in a naïve population while considering the public demand for community-level control and, importantly, the effects of personal-level protection on population-level outbreak dynamics. We consider scenarios at low, medium, and high levels of community-level vector control, and at each level, we consider combinations of low, medium, and high levels of motivation to use personal protection, namely concern for disease transmission and concern for being bitten in general.

**Results:**

When there is very little community-level vector control, nearly the entire population is quickly infected, regardless of personal protection use. When vector control is at an intermediate level, both concerns that motivate the use of personal protection play an important role in reducing disease burden. When authorities have the capacity for high-level community vector control through pesticide use, the motivation to use personal protection to reduce disease transmission has little additional effect on the outbreak.

**Conclusions:**

While results show that personal-level protection alone is not enough to significantly impact an outbreak, personal protective measures can significantly reduce the severity of an outbreak in conjunction with community-level control. Furthermore, the model provides insight for targeting public health messaging to increase the use of personal protection based on concerns related to being bitten by mosquitos or vector-borne disease transmission.

**Graphical Abstract:**

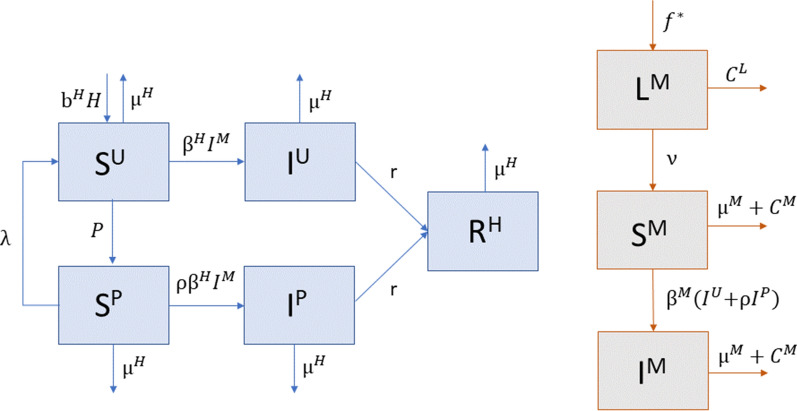

**Supplementary Information:**

The online version contains supplementary material available at 10.1186/s13071-022-05481-7.

## Background

Mosquito-borne disease outbreaks pose a significant public health threat, particularly with the looming threat of climate change yielding a wider range of suitable habitats for mosquito populations to thrive. Climate variability and extreme weather events associated with climate change are expected to result in global increases in the frequency and severity of mosquito-borne diseases [1–4]. In addition, expanding urbanization, international travel and trade, and population growth contribute to the potential for the introduction and sustainability of a mosquito-borne disease outbreak in a naïve population [5, 6]. As public health authorities have little control of these underlying factors, they can expect to face an expanding spectrum of vector-borne diseases with increasing incidence and geographical spread [5, 7].

Successful control of a mosquito-borne disease outbreak requires timely, multi-pronged efforts as well as coordination across sub-populations [8–12]. Effective vector control relies on existing public health infrastructure, such as surveillance of vector populations (size, disease incidence), to understand and prepare for the risk of impending mosquito-borne disease outbreaks. Common interventions include the use of larvicides and adulticides to reduce vector populations and targeted communications to increase public awareness regarding the need for personal protective measures, such as usage of mosquito repellant, protective clothing, and mosquito nets [1]. Community vector control in the USA consists of patchwork operations with wide variability in capacity for control activities; in a recent study of over 1000 vector-control organizations, 84% were found critically lacking in capacity for essential vector-control operations [5]. These shortfalls pose significant challenges when trying to enact coordinated community-level vector control efforts sufficient to mitigate the risks of an outbreak.

Broad-scale mosquito control programs are increasingly difficult to implement and maintain because of financial and environmental considerations and the potential for increasing insecticide resistance. Mitigation efforts may therefore be more successful by emphasizing the use of personal protective measures [6, 13]. Public health surveys in areas with endemic mosquito-borne diseases show that most people within the study communities perceived mosquitos as a problem, as both a biting nuisance (not related to disease) and vectors of human disease [14, 15]. Another public health survey found the odds of personal protection use to be significantly associated with awareness, perceived susceptibility, and perceived severity of the mosquito-borne diseases included in the survey [13]. These community surveys highlight the importance of public health campaigns focused on increasing public knowledge, particularly in these three key areas.

Previous works have used mathematical models to explore effective vector control strategies, including timeliness of implementation [11], community coordination [8, 9], and trade-offs between vector control and environmental concerns [16, 17]. In each of these model formulations, control is only considered as a population-level approach and does not consider individual-level personal protection. In addition, many vector-borne disease models that include multiple host classes to represent personal protection assume a static assignment of individuals to protected and unprotected classes [18–22].

Here, we propose a simplified compartmental model of mosquito-borne disease dynamics that incorporates the use of personal protection against mosquito bites considering two key individual-level behavioral drivers of use—concern for being bitten by mosquitos as a nuisance and concern for mosquito-borne disease transmission. Individual-level behavior fluctuates over the course of the outbreak, as individuals continuously assess their protection status based on the adult mosquito population size and the prevalence of infected humans. We also consider aggregate average public demand for local-level vector control by authorities in our model, which is also dictated by the infected human population. This general framework can be used to understand the motivations for, and impact of, individual choices to use personal protection, which can further improve decisions in public health campaigns and ultimately reduce burden from vector-borne diseases.

## Methods

### Model

The modified Ross-Macdonald model presented in Suarez [16] describes the dynamics of vector-borne disease spread in a naïve population while considering the public demand for control and the environmental concern the public may have regarding broad use of pesticides [16, 23–25]. Their model reflects the specific case of Zika virus in *Aedes aegypti* mosquitos. We propose a modified version of the model to include the effects of personal-level protection on population-level mosquito-borne disease dynamics.

**Fig. 1:**
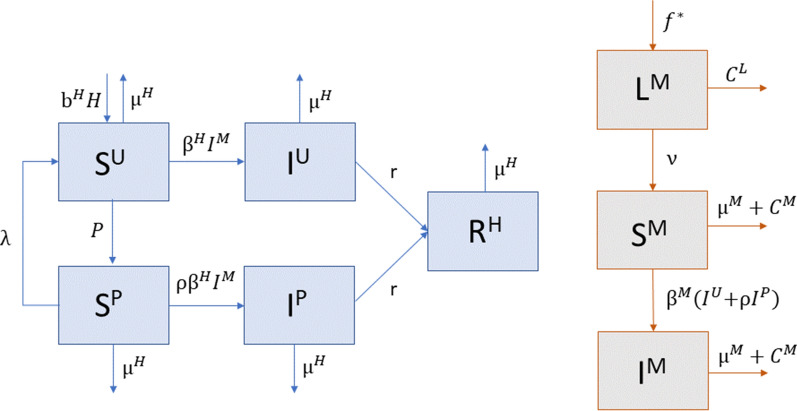
Visual representation of the human (blue) and mosquito (gray with orange outline) population dynamics in an isolated patch, where infected humans (I^U^, I^P^) infect susceptible mosquitos (S^M^), and infected mosquitos (I^M^) infect susceptible humans (S^U^, S^P^). The mosquito and larval control variables (*C*^M^ and *C*^L^ respectively) are not shown as compartments here

In this model, humans in the population are characterized as susceptible (S^H^), infected (I^H^), or recovered (R^H^), where the susceptible and infected populations are further divided into protected (S^P^, I^P^) and unprotected (S^U^, I^U^) classes, as shown in Fig 1. Humans in the two protected classes use personal protective measures that protect against contact with mosquitos at an assumed 80% efficacy; therefore, the transmission rates from infected mosquito to susceptible human (*β*^H^) and from infected human to susceptible mosquito (*β*^M^) are reduced by 80% via the parameter *ρ* = 0.2 in the protected classes. For simplicity, we do not consider vertical transmission in either the host or vector or human-to-human sexual transmission of the virus.

We assume that susceptible individuals continuously re-assess their use of personal protection and move from the unprotected susceptible class to the protected susceptible class based on the overall motivation to use personal protection ($$P)$$, where they remain protected for 2 days (1/*λ*) before returning to the unprotected class (and can then decide anew to use protection). In this model, humans are motivated to use personal protection by two separate objectives, to reduce disease transmission and to reduce biting as a general nuisance. The use of personal protection to reduce disease transmission is motivated by the concern of disease ($${\upgamma }^{\text{D}}$$) and the number of infections in the population, whereas the motivation related to reducing bites is driven by the concern of biting ($${\upgamma }^{\text{B}}$$) and the size of the adult mosquito population.

For simplicity, we assume that protected individuals who become infected remain protected through the infectious period, and those who are unprotected at the time of infection remain unprotected; thus, protected susceptible humans move into the protected infectious class, and unprotected susceptible humans move into the unprotected infectious class (Fig. 1). This ensures that humans are not bouncing back and forth between the two infectious compartments without recovering. Individuals in both infectious classes move to the recovered class at rate *r*. Humans in the recovered class develop immunity and do not return to the susceptible class, so an individual cannot be infected more than once. We also include natural births and deaths at rates *b*^H^ and *μ*^H^, respectively.

In keeping with the Suarez et al. model [16], we consider the spread of disease related to local human mobility. We represent the spatial distribution of the population as a square matrix with *N* patches, where patches can be considered as average city blocks in a US suburban area. Individuals in the population can randomly move from any one patch to any other patch. The flux of people moving from patch *i* to patch *j* is expressed as *m*_*ij*_, where *m*_*ii*_ = 0, and ${\sum }_{j=1}^{N}{m}_{ij}$ = *p* and *p* is the probability of traveling.

The mosquito population is divided into three classes: juvenile mosquitos, or larvae, that do not transmit the virus (L^M^), uninfected adult mosquitos that are susceptible to the virus (S^M^), and infected adult mosquitos that can transmit the virus by biting a susceptible human (I^M^) (Fig. 1). Adult mosquitos lay eggs at rate $\eta$, and each patch has a limited carrying capacity for larvae, which is defined by the function $f\left({M}_{j},{K}_{j}\right)={M}_{j}(1-{M}_{j}/{K}_{j})$, where ${M}_{j}={S}_{j}^{M}+{I}_{j}^{M}$ is the number of adult mosquitos that can lay eggs, and ${K}_{j}$ is the maximum carrying capacity for patch *j*. The larvae transition to the susceptible adult class at rate *ν*, and adult mosquitos die at rate *μ*^M^. In addition, mosquitos in the model remain within the patch they were born based on evidence that *Aedes egypti* rarely travel large distances [26–28]; therefore, the spatial spread of the virus across patches relies solely on human mobility.

Humans can also demand for authorities to enact mosquito control measures, such as larvicides and adulticides. The amount of control measures applied in a given patch is assumed to be equivalent to the demand, and it increases with the number of infected people in the patch [16]. The demand for adult mosquito control (*C*^M^) and the demand for larvae control (*C*^L^) are also impacted by environmental concern regarding the use of pesticides ($\epsilon$). The use of large amounts of pesticides can produce concern over the perceived risks and potential side effects, even if the pesticides used are innocuous, discouraging the demand for control. Thus, we assume a linear dependence between the quantity of pesticides applied and the level of environmental concern in the patch [16].

For a given patch *j*,

$$\begin{aligned} \frac{{d{\text{S}}_{j}^{{\text{U}}} }}{dt} & = b^{{\text{H}}} H_{j} - \beta^{{\text{H}}} {\text{I}}_{j}^{{\text{M}}} {\text{S}}_{j}^{{\text{U}}} - P_{j} {\text{S}}_{j}^{{\text{U}}} + \lambda {\text{S}}_{j}^{{\text{P}}} - \mu^{{\text{H}}} {\text{S}}_{j}^{{\text{U}}} + \mathop \sum \limits_{i} m_{ij} {\text{S}}_{i}^{{\text{U}}} - {\text{S}}_{j}^{{\text{U}}} \mathop \sum \limits_{i} m_{ji} \\ \frac{{d{\text{S}}_{j}^{{\text{P}}} }}{dt} &= P_{j} {\text{S}}_{j}^{{\text{U}}} - \lambda {\text{S}}_{j}^{{\text{P}}} - \mu^{{\text{H}}} {\text{S}}_{j}^{{\text{P}}} - \rho \beta^{{\text{H}}} {\text{I}}_{j}^{{\text{M}}} {\text{S}}_{j}^{{\text{P}}} + \mathop \sum \limits_{i} m_{ij} {\text{S}}_{i}^{{\text{P}}} - {\text{S}}_{j}^{{\text{P}}} \mathop \sum \limits_{i} m_{ji} \\ \frac{{d{\text{I}}_{j}^{{\text{U}}} }}{dt} & = \beta^{{\text{H}}} {\text{I}}_{j}^{{\text{M}}} {\text{S}}_{j}^{{\text{U}}} - r{\text{I}}_{j}^{{\text{U}}} - \mu^{{\text{H}}} {\text{I}}_{j}^{{\text{U}}} + \mathop \sum \limits_{i} m_{ij} {\text{I}}_{i}^{{\text{U}}} - {\text{I}}_{j}^{{\text{U}}} \mathop \sum \limits_{i} m_{ji} \\ \frac{{d{\text{I}}_{j}^{{\text{P}}} }}{dt}& = \rho \beta^{{\text{H}}} {\text{I}}_{j}^{{\text{M}}} {\text{S}}_{j}^{{\text{P}}} - r{\text{I}}_{j}^{{\text{P}}} - \mu^{{\text{H}}} {\text{I}}_{j}^{{\text{P}}} + \mathop \sum \limits_{i} m_{ij} {\text{I}}_{i}^{{\text{P}}} - {\text{I}}_{j}^{{\text{P}}} \mathop \sum \limits_{i} m_{ji} \\ \frac{{d{\text{R}}_{j}^{{\text{H}}} }}{dt} & = r{\text{I}}_{j}^{{\text{U}}} + r{\text{I}}_{j}^{{\text{P}}} - \mu^{{\text{H}}} {\text{R}}_{j}^{{\text{H}}} + \mathop \sum \limits_{i} m_{ij} {\text{R}}_{i}^{{\text{H}}} - {\text{R}}_{j}^{{\text{H}}} \mathop \sum \limits_{i} m_{ji} \\ \frac{{d{\text{L}}_{j}^{{\text{M}}} }}{dt} & = f\left( {\eta \left( {{\text{S}}_{j}^{{\text{M}}} + {\text{I}}_{j}^{{\text{M}}} } \right),K_{j} } \right) - \nu {\text{L}}_{j}^{{\text{M}}} - C_{j}^{{\text{L}}} {\text{L}}_{j}^{{\text{M}}} \\ \frac{{d{\text{S}}_{j}^{{\text{M}}} }}{dt} &= \nu {\text{L}}_{j}^{{\text{M}}} - \beta^{{\text{M}}} {\text{I}}_{j}^{{\text{U}}} {\text{S}}_{j}^{{\text{M}}} - \rho \beta^{{\text{M}}} {\text{I}}_{j}^{{\text{P}}} {\text{S}}_{j}^{{\text{M}}} - \mu^{{\text{M}}} {\text{S}}_{j}^{{\text{M}}} - C_{j}^{{\text{M}}} {\text{S}}_{j}^{{\text{M}}} \\ \frac{{d{\text{I}}_{j}^{{\text{M}}} }}{dt} & = \beta^{{\text{M}}} {\text{I}}_{j}^{{\text{U}}} {\text{S}}_{j}^{{\text{M}}} + \rho \beta^{{\text{M}}} {\text{I}}_{j}^{{\text{P}}} {\text{S}}_{j}^{{\text{M}}} - \mu^{{\text{M}}} {\text{I}}_{j}^{{\text{M}}} - C_{j}^{{\text{M}}} {\text{I}}_{j}^{{\text{M}}} \\ \frac{{dC_{j}^{{\text{M}}} }}{dt} & = \gamma^{C} {\text{I}}_{j}^{{\text{H}}} - \epsilon C_{j}^{{\text{M}}} \\ \frac{{dC_{j}^{{\text{L}}} }}{dt} & = \gamma^{{\text{C}}} {\text{I}}_{j}^{{\text{H}}} - \epsilon C_{j}^{{\text{L}}} \\ \end{aligned}$$

where ${H}_{j}={\text{S}}_{j}^{\text{U}}+{\text{S}}_{j}^{\text{P}} +{\text{I}}_{j}^{\text{U}} +{\text{I}}_{j}^{\text{P}}+{\text{R}}_{j}^{\text{H}}$ is the human population size, ${\text{I}}_{j}^{\text{H}}={\text{I}}_{j}^{\text{U}}+{\text{I}}_{j}^{\text{P}}$ is the total number of infectious humans, and ${P}_{j}=\left({\upgamma }^{\text{D}}\frac{{\text{I}}_{j}^{\text{H}}}{{H}_{j}}+{\upgamma }^{\text{B}}{M}_{j}\right)$ represents the motivation to use personal protection based on the proportion of infected individuals and the number of mosquitos in the patch. The model variables are defined in Table 1, and model parameter descriptions and values are presented in Table 2.

**Table 1:** Model variables and initial conditions for each patch

Variable	Description	Initial value
${\text{S}}_{j}^{\text{U}}$	Unprotected susceptible humans	$\in [700, 800]$
${\text{S}}_{j}^{\text{P}}$	Susceptible humans using personal protection	0
${\text{I}}_{j}^{\text{U}}$	Unprotected infectious humans	1 in a random patch
${\text{I}}_{j}^{\text{P}}$	Infectious humans using personal protection	0
${\text{R}}_{j}^{\text{H}}$	Recovered humans	0
${\text{L}}_{j}^{\text{M}}$	Mosquito larvae	0
${\text{S}}_{j}^{\text{M}}$	Susceptible mosquitos	$\in [\mathrm{1200,1300}]$
${\text{I}}_{j}^{\text{M}}$	Infectious mosquitos	0
${C}_{j}^{\text{M}}$	Mosquito control	0
${C}_{j}^{\text{L}}$	Larvae control	0

The initial number of unprotected susceptible humans and susceptible mosquitos for each patch is selected from a uniform distribution within the specified interval

**Table 2:** Model parameters and their associated values for the scenarios presented

Parameter	Description	Value	References
${\beta }^{H}$	Transmission rate for humans	1.5 × 10^–4^	Assumed
${\beta }^{M}$	Transmission rate for mosquitos	3.0 × 10^–4^	Assumed
$\uprho$	Relative transmission for protected humans	0.2	Assumed
${\gamma }^{\text{D}}$	Concern of disease transmission [low, medium, high]	[15, 150, 1500]/700	Assumed
${\gamma }^{\text{B}}$	Concern of being bitten [low, medium, high]	[0.1, 0.5, 1]/1200	Assumed
$1/\lambda$	Average length of use of personal protection for susceptible humans	2	Assumed
${\mu }^{\text{H}}$	Natural death rate for humans	(8.6/1000)/365	[9]
${\mu }^{\text{M}}$	Natural death rate for mosquitos	1/13	[9]
${\text{b}}^{\text{H}}$	Human birth rate	(9/1000)/365	[30]
$r$	Human recovery rate	0.037	[31]
$\nu$	Maturation rate (larvae to mosquito)	1/7	[9]
$\eta$	Mosquito egg laying rate	10	[9]
$p$	Fraction of people that travel between patches	0.2	[16]
$\epsilon$	Environmental concern that demotivates pesticide usage for mosquito and larval control [low, medium, high]	[500, 200, 150]	[16]
${\upgamma }^{\text{C}}$	Demand for community level vector control influenced by disease in the population	${e}^{-\upepsilon /50}$	[16]
$\tau$	Time delay on application of control	7	Assumed
${K}_{j}$	Larvae carrying capacity for each patch (*j*)	$\in [20000, 25000]$	[16]
$N$	Number of patches	25	

We assume the same constant values for each patch, excluding the larvae carrying capacity which can vary across patches. The assumed rates were chosen to reflect early Zika outbreaks. Each rate is presented on a daily timescale

### Scenarios

To analyze the impact of personal-level protection on population-level outbreak dynamics, we consider scenarios at low, medium, and high levels of community-level vector control (${\upgamma }^{\text{C}})$ by authorities. At each level, we consider combinations of the personal protection parameters with low, medium, and high levels of concern of disease transmission (${\upgamma }^{\text{D}}$) and of concern of being bitten in general (${\upgamma }^{\text{B}}$). For each scenario, we solve the system of differential equations using an explicit Runge-Kutta formula [29], and then we compare outbreak characteristics across the 27 different parameter combinations.

## Results

We present scenarios with low, medium, and high levels of concern for disease transmission (${\gamma }^{\text{D}}$) and concern for being bitten in general (${\gamma }^{\text{B}}$) to compare outcomes across scenarios. We consider each combination of these two parameters at low, medium, and high levels of community vector control through larvicides and adulticides (Table 2).

In addition, we analyze the sensitivity of parameters with semi-arbitrary values, including the length of protection (*λ*) and the probability of mobility (*p*). We present an abridged version in the Results with more details included within Additional file 1 (Additional file 1: Figs. S1-S2). We also include figures that show the vector dynamics for each level of community control within Additional file 1 to show how the vector population changes with level of control and proportion of humans infected in the population (Additional file 1: Figs. S3–S5).

### Low community-level control

**Fig. 2:**
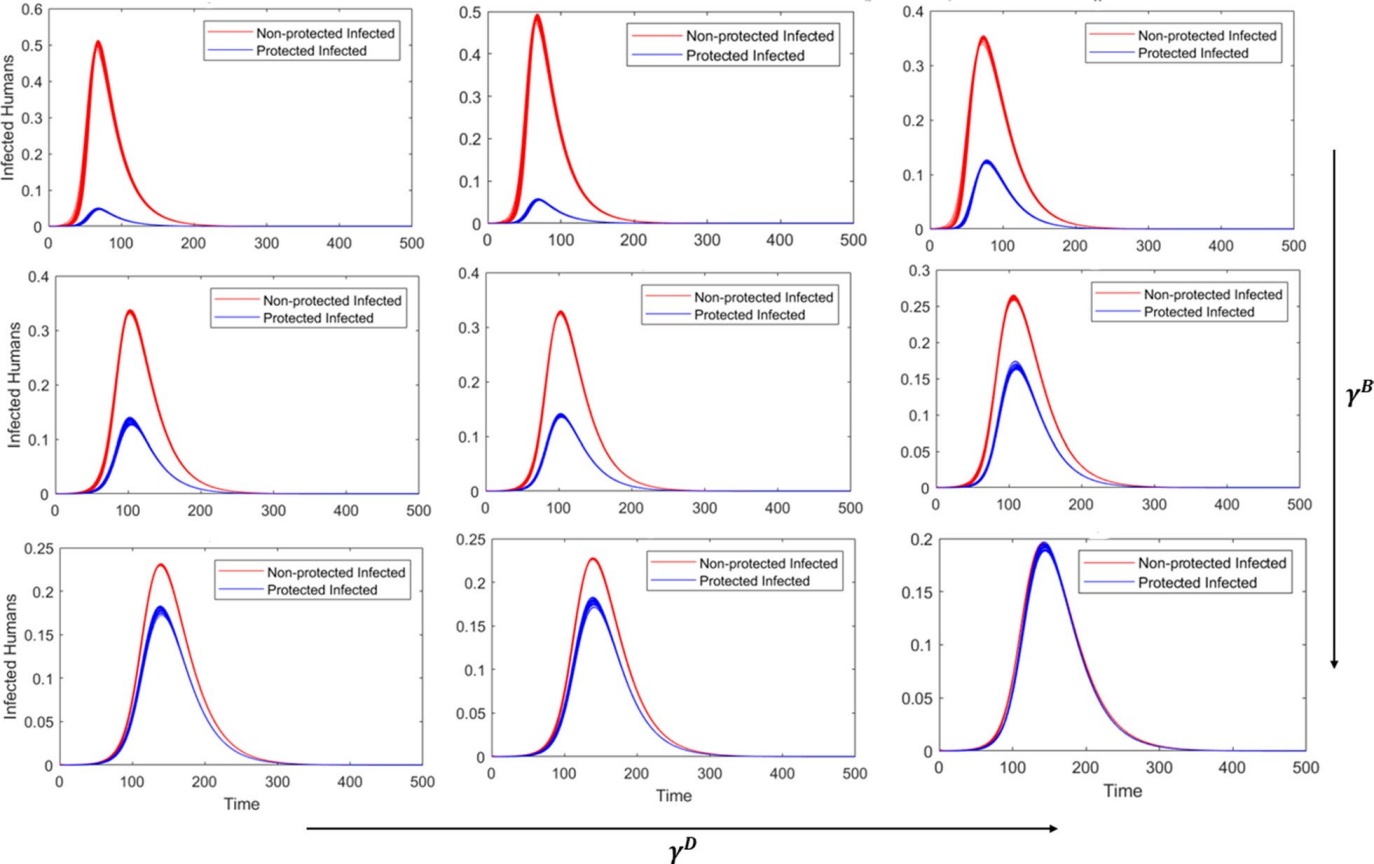
Incidence curves for each of the simulated scenarios with low community-level vector control, where red curves represent the non-protected infectious proportion of the population and blue represent the proportion of protected infections. The level of concern related to biting (${\gamma }^{\mathrm{B}})$ increases as you move down columns, and the concern of disease (${\gamma }^{\text{D}})$ increases as you move across the rows. High, medium, and low values of ${\gamma }^{\text{B}}$ and ${\gamma }^{\text{D}}$ are presented in Table 2

When there is very little to no patch-level vector control by authorities, nearly the entire population is quickly infected, regardless of personal protection use (Fig. 2). Higher levels of concern for being bitten are correlated with lower peaks that occur later in the epidemic, and increasing the concern for disease yields slightly lower peaks with little-to-no difference in peak timing. The differences in peak timing, peak size, and epidemic length are noted; however, the practical differences are not likely significant, as > 95% of the population is infected by the end of each scenario (Table 3).

**Table 3:** Final proportion of population infected by the end of the outbreak for each of the nine scenarios with low community-level control

	Low ${\gamma }^{\text{D}}$	Medium ${\gamma }^{\text{D}}$	High ${\gamma }^{\text{D}}$
Low ${\gamma }^{\text{B}}$	99.68%	99.10%	98.87%
Medium ${\gamma }^{\text{B}}$	98.67%	98.18%	97.56%
High ${\gamma }^{\text{B}}$	96.94%	96.21%	95.74%

### Medium community-level control

**Fig. 3:**
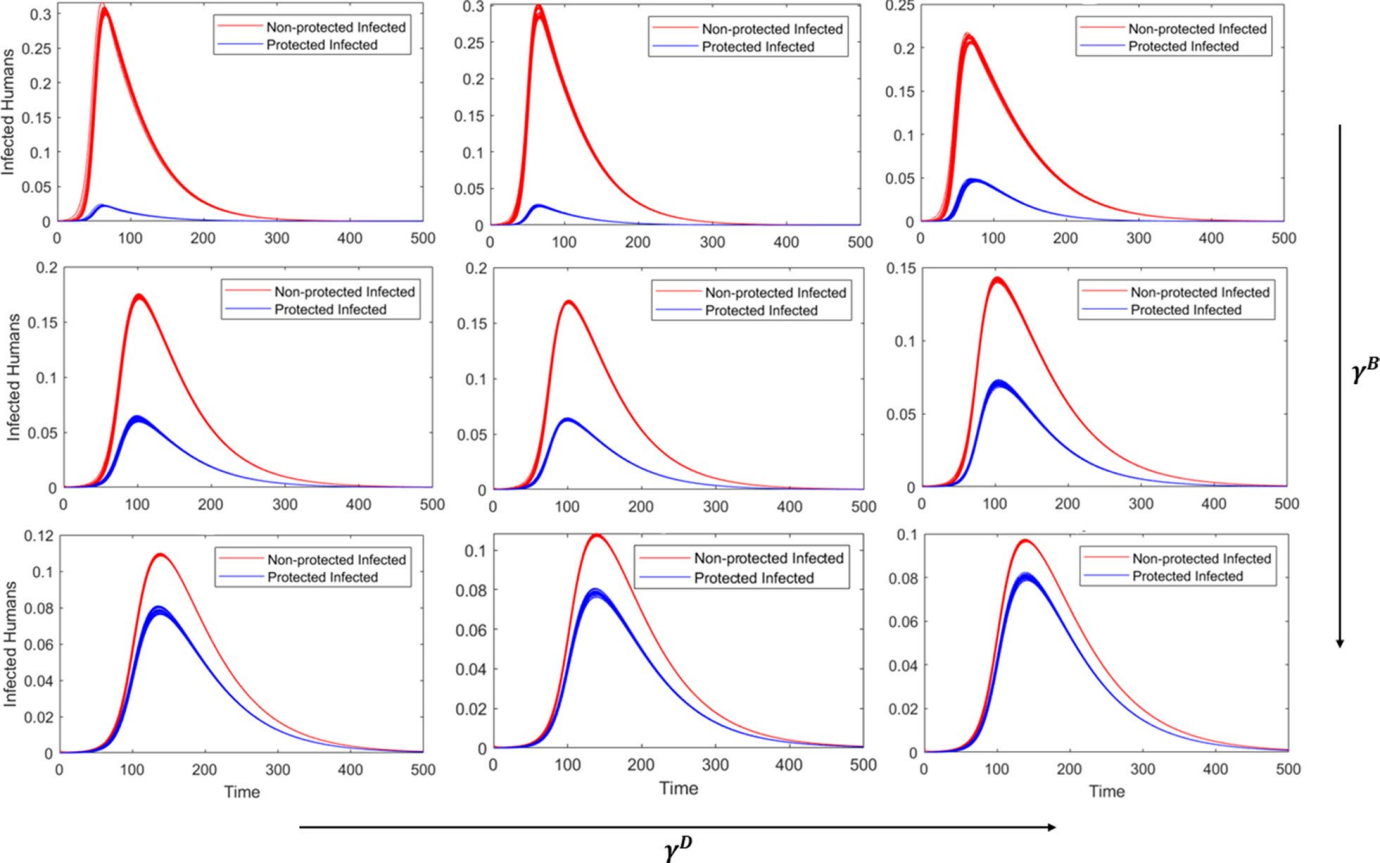
Incidence curves for each of the simulated scenarios with medium level community-level vector control, where red curves represent non-protected infections and blue represent protected infections. The level of concern related to biting (${\gamma }^{\mathrm{B}})$ increases as you move down columns, and the concern of disease (${\gamma }^{\text{D}})$ increases as you move across the rows. High, medium, and low values of ${\gamma }^{\mathrm{B}}$ and ${\gamma }^{\text{D}}$ are presented in Table 2

At intermediate or medium levels of vector control, we observe the most interesting dynamics of personal protection. At low concern for disease (Fig. 3, first column), increasing concern for being bitten extends the length of the outbreak but does not impact the final outbreak size, with > 93% of the population infected by the end of each simulation (Table 4). Likewise, when the level of concern for bites is low (Fig. 3, first row), personal protection for concern of disease has little impact on the outbreak, with small differences in final proportion infected (Table 4).

**Table 4:** Final proportion of population infected by the end of the outbreak for each of the nine scenarios with medium-level community control

	Low ${\gamma }^{\text{D}}$	Medium ${\gamma }^{\text{D}}$	High ${\gamma }^{\text{D}}$
Low ${\gamma }^{\text{B}}$	93.53%	90.71%	88.57%
Medium ${\gamma }^{\text{B}}$	93.53%	79.98%	77.72%
High ${\gamma }^{\text{B}}$	93.53%	67.92%	66.20%

While the dynamics visually look similar across ${\gamma }^{\text{D}}$ (within rows, Fig. 3), there is a significant decrease in the number of infections comparing low concern for disease to medium or high, particularly for medium and high ${\gamma }^{\text{B}}$ (Table 4). At medium levels of ${\gamma }^{\text{B}}$, for example, the outbreak curves look similar, while the total proportion infected decreases from 93.53% to 79.98% as ${\gamma }^{\text{D}}$ increases from low to medium (Table 4). There is little difference, however, between medium and high ${\gamma }^{\text{D}}$. In addition, protection use to reduce bites significantly lowers the total proportion infected from 0.91 to 0.8 to 0.68 (medium ${\gamma }^{\text{D}}$) per level of ${\gamma }^{\text{B}}$ (Table 4).

### High community-level control

**Fig. 4:**
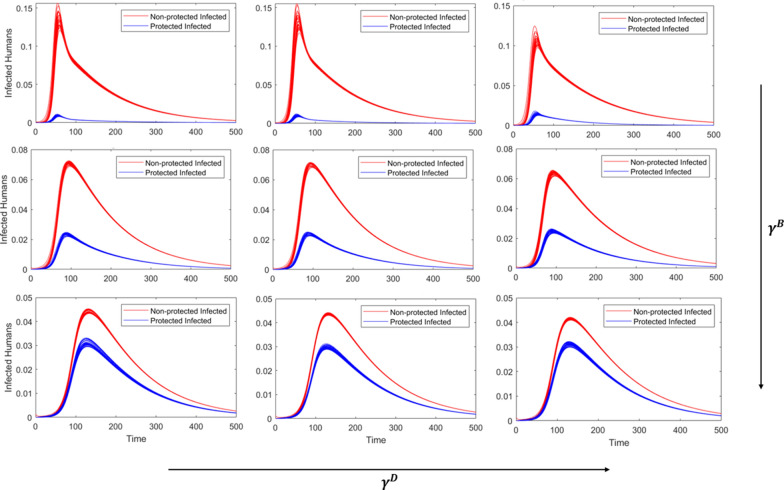
Incidence curves for each of the simulated scenarios with high-level community vector control, where red curves represent non-protected infections and blue represent protected infections. The level of concern related to biting (${\gamma }^{\mathrm{B}})$ increases as you move down columns, and the concern of disease (${\gamma }^{\text{D}})$ increases as you move across the rows. High, medium, and low values of ${\gamma }^{\mathrm{B}}$ and ${\gamma }^{\text{D}}$ are presented in Table 2

When the population demand for vector control is high, increasing concern for disease transmission yields little differences while holding ${\gamma }^{\text{B}}$ constant (within rows, Fig. 4); however, this is largely due to the formulation of the equations influencing both community- and personal-level control. The use of personal protection to reduce disease transmission (${\gamma }^{\text{D}}\frac{{\text{I}}^{\text{H}}}{H}$) and the demand for community-level control (${\gamma }^{\mathrm{C}}{\mathrm{I}}^{\mathrm{H}})$ both increase with the number of infected individuals, and community control impacts the dynamics at a greater magnitude than personal protection, such that higher levels of concern for disease transmission have little impact on the overall dynamics. The general concern for being bitten, however, significantly reduces the outbreak peaks and total proportion of the population that becomes infected for each level of ${\gamma }^{\text{D}}$ (Fig. 4, Table 5).

**Table 5:** Final proportion of population infected by the end of the simulation for each of the nine scenarios with high-level community control

	Low ${\gamma }^{\text{D}}$	Medium ${\gamma }^{\text{D}}(\mathrm{\%})$	High ${\gamma }^{\text{D}}$
Low ${\gamma }^{\text{B}}$	58.29%	58.28%	56.82%
Medium ${\gamma }^{\text{B}}$	45.08%	45.19%	43.98%
High ${\gamma }^{\text{B}}$	33.96%	33.56%	33.16%

In addition, it should be noted that the scenarios for high community-level control differ from low and medium in that the number of infections do not reach zero by the end of the 500-day simulations (Fig. 4). The dynamics suggest that introduction of an infected individual would result initially in a spike of cases that dwindles over time, resulting in long tails at low infection levels and suggesting the outbreaks will continue beyond this point.

### Sensitivity analyses

Within the model, individuals continuously reassess their use of personal protection based on both the concern for disease and the concern for being bitten, which are influenced by the proportion of infected humans in the population and the number of mosquitos, respectively. When motivation is high enough, individuals move into the protected class, where they remain for 2 days (1/*λ*) before moving back to the unprotected class to reassess their motivation to be protected. We semi-arbitrarily chose the length of 2 days, as we assumed a shorter time period would be more realistic than assuming individuals are constantly protected for longer periods of time. However, we were interested in observing how the resulting dynamics of the simulations may change for different values of *λ*.

We ran many scenarios for increasing lengths of protection to assess how the dynamics change as *λ* increases. The results show that increasing the length of time in the protected class results in a higher percentage of susceptible individuals being protected (Additional file 1: Fig. S1). This is intuitive, as individuals stay in the class longer, meaning more individuals will enter the class before they leave to reassess their status. This further results in more protected infections, as the majority of the population is protected, and fewer overall infections, as protection plays a role in reducing new infections in both humans and mosquitos. We note that this parameter could be utilized to distribute susceptible individuals between protected and unprotected classes to adjust the initial conditions to mirror the proportions within the study population.

We also analyzed the sensitivity of the dynamics to the value of *p*, or the probability of individuals moving between patches. The only notable trend across increasing levels of *p* is the existence of more variability between patches for a low *p* = 0.1 (Additional file 1: Fig. S2). In the first row of Additional file 1: Fig. S2, we can observe differences in outbreak timing across patches, represented by the staggered start of the individual lines, as opposed to the nearly perfectly overlapping curves for the higher values of *p*. Other than this difference for very low values of *p*, our results show that increasing the value of *p* has little additional impact on the dynamics.

## Discussion

The explored scenarios provide actionable insight for effective public health messaging in combatting the threat of vector-borne diseases. First, our results support that vector control through pesticide use by authorities is likely to be the most effective strategy for reducing disease burden and that personal protection measures cannot effectively constrain the outbreak when local vector control is not enacted. However, when vector control is available, the use of personal protective measures is an effective tool for further reducing disease burden.

When vector control is at an intermediate level, both concerns that motivate the use of personal protection play an important role in reducing disease burden. For medium and high levels of concern for disease, concern for bites clearly reduces the total number of infections (Table 4). When the concern for disease is low, the use of protection to prevent bites has no impact on the total number of infections (Table 4); it does, however, increase the length of the outbreak and lower the size of the peak, essentially ‘flattening the curve’ (Fig. 3). Therefore, based on our specific parameters and simulation scenarios, the most effective public health messaging approach would ensure messaging focused on both vector-borne disease transmission as well as general mosquito bite prevention.

When authorities have the capacity for high-level community vector control through pesticide use, the motivation to use personal protection to reduce disease transmission has little additional effect on the outbreak. The motivation to use personal protection to reduce disease transmission and the motivation to demand community-level control are both dictated by the disease incidence, so at a high level of demand for pesticide use, the personal protection makes little difference on the outbreak dynamics (essentially, community-level control dwarfs the impact from personal protection). However, the general concern for being bitten still significantly impacts the total proportion infected (Table 5), as mosquito bite prevention provides earlier intervention compared to reacting to already-occurring disease incidence. Therefore, in these presented scenarios, public health outreach should focus on general seasonal protection from mosquitos rather than disease prevention. When demand for control is already high, it may be a waste of resources to focus additional outreach on preventing disease transmission.

We emphasize that the presented results refer to the specific simulations explored within this paper and thus may not be generalizable across all settings. We also reiterate that the presented model is a simplified representation of more complex mosquito-borne disease dynamics; as such, the results should be interpreted with relation to the assumptions required. For example, our model assumes that the community-level control is 100% effective; however, this is unlikely representative of actual vector control operations, and decreasing community-level efficacy could then increase the relative impact of bite avoidance. The model does not consider aspects that can negatively impact the efficacy of vector control, like insecticide resistance or improper choice of insecticide. Our model also does not consider the seasonality of mosquito biting, so mosquitos are assumed to bite equally frequently all year, which is not representative of the patterns observed in nature. Incorporating the fluctuation of mosquito biting habits with seasonal changes may provide more realistic results for long-term scenarios and would potentially identify phases of vector ecology during which to tailor specific foci for public health messaging (i.e., when to highlight disease transmission versus bite avoidance).

Another simplifying assumption in our model is that each patch in the population reacts equivalently regarding vector control and environmental concern. In addition, each patch is considered at the same risk which is likely an unreasonable assumption. It would be interesting for future research to explore disparities in mosquito-borne disease risk and how targeted messaging vs. general messaging may impact the dynamics—particularly because typically disadvantaged areas with higher risk of an outbreak will also have lower access to health care as well as less disposable income to spend on personal repellent and protective clothing.

## Conclusions

Overall, our model scenarios show the importance of personal protection use as a vital mitigation strategy for mosquito-borne disease outbreaks. While personal-level protection alone is not enough to significantly impact an outbreak, personal protective measures can significantly reduce the total infections in conjunction with community-level control. Furthermore, public health messaging can be targeted to increase the use of personal protection based on concerns related to being bitten by mosquitos or vector-borne disease transmission. Some scenarios benefit from a two-pronged messaging approach including general mosquito bite prevention as well as disease transmission prevention, while other scenarios show that focusing attention on disease transmission may not be cost-effective or significantly reduce disease burden. Therefore, public health officials must consider the concerns of the population to provide effective messaging that encourages personal protection use to manage risks from a mosquito-borne disease.

## Supplementary Information

**Additional file 1.** Supplementary file containing an expanded description of the sensitivity analyses (with figures) and additional figures with vector population dynamics.

## Data Availability

Data sharing is not applicable to this article as no datasets were generated or analyzed during the current study.
