# Supplementary material for: A general modeling framework for exploring the impact of individual concern and personal protection on vector-borne disease dynamics
Source: Parasit Vectors. 2022 Oct 8;15:361. doi: 10.1186/s13071-022-05481-7 (PMC9548150; doi:10.1186/s13071-022-05481-7)
Supplement: Supplementary file 1 — Additional file 1. Supplementary file containing an expanded description of the sensitivity analyses (with figures) and additional figures with vector population dynamics. [file 13071_2022_5481_MOESM1_ESM.docx]

**Additional file 1**

*Sensitivity analyses*

*Length of personal protection, λ*

As discussed in the introduction, many existing models that assess individual-level protection include static assignment of individuals, meaning they are classified as either protected or not and remain that way throughout the outbreak. For our model, we wanted to allow individuals to continuously reassess their protection status and move between protected and unprotected classes, as we believe these dynamics would be closer to reality. Motivation to use personal protection is based on both the concern for disease (γ^D^) and concern for being bitten (γ^B^), which are influenced by the proportion of infected humans in the population and the number of mosquitos, respectively. When motivation is high enough, individuals move into the protected class, where they remain for 2 days (1/λ) before moving back to the unprotected class to once again assess their motivation to be protected.

This parameter λ, the rate from the protected susceptible class to the unprotected susceptible class, was semi-arbitrarily chosen to represent a short time period (2 days) that one remains protected. We kept this low, as we thought it unrealistic to assume every individual using personal protection would do so for the entirety of a week, for example. However, we were interested in how changing this parameter may impact the dynamics observed.

We ran many scenarios for increasing lengths of protection to assess how the dynamics change as 1/λ increases. The results show that increasing the length of time in the protected class results in a higher percentage of susceptible individuals being protected (Figure S1). This is intuitive, as individuals stay in the class longer, meaning more individuals will enter the class before those leave to reassess their status. This further results in more protected infections, as the majority of the population is protected, and fewer overall infections, as protection plays a role in reducing new infections in both humans and mosquitos.

Figure S1 shows the results of increasing 1/ λ for the scenario with high community control, and medium concerns for biting and disease transmission. We also ran this for many other scenarios, with results being comparable for each, excluding scenarios with low community-level control, where it makes little difference on the dynamics. This is in line with our results in the main text that personal protection has little effect on the dynamics when community control is low; thus increasing the length of protection would also have little effect. It should be noted that this parameter could be utilized to distribute susceptible individuals between protected and unprotected classes to mirror the proportions in a population with the model.

*Probability of movement between patches, p*

We also analyzed the sensitivity of the dynamics to the value of *p*, or the probability of moving between patches; essentially, increasing *p* increases the movement of humans. The only notable trend across increasing levels of *p* is the existence of more variability between patches for a low *p=*0.1 (Figure S2). In the first row of Figure S2, we can actually observe differences in outbreak timing across patches, represented by the staggered start of the individual lines, as opposed to the nearly perfectly overlapping curves for the higher values of *p*. Other than for very low values of *p*, our results show that it has little additional impact on the dynamics.

This is in line with the findings of the Suarez model (1), where sensitivity analyses also showed that dynamics with *p* 🡪 0 are very similar to scenarios with larger *p*, just shifted in time. The shift in time is also shown in our figure for the lowest value of *p*. In addition, the sensitivity analyses in (1) showed that as p 🡪 0, it takes longer for the disease to reach neighboring patches; however, the outbreak can still reach all patches, indicating that regardless how little movement exists, the disease will still manage to spatially spread within a naïve population.

**Figure S1.** Dynamics of the susceptible and infectious populations for increasing lengths of protection (1/λ), keeping all other parameter values the same, for scenarios with high community control, and medium concerns for biting and disease transmission. Blue curves represent protected individuals, while red represents unprotected.


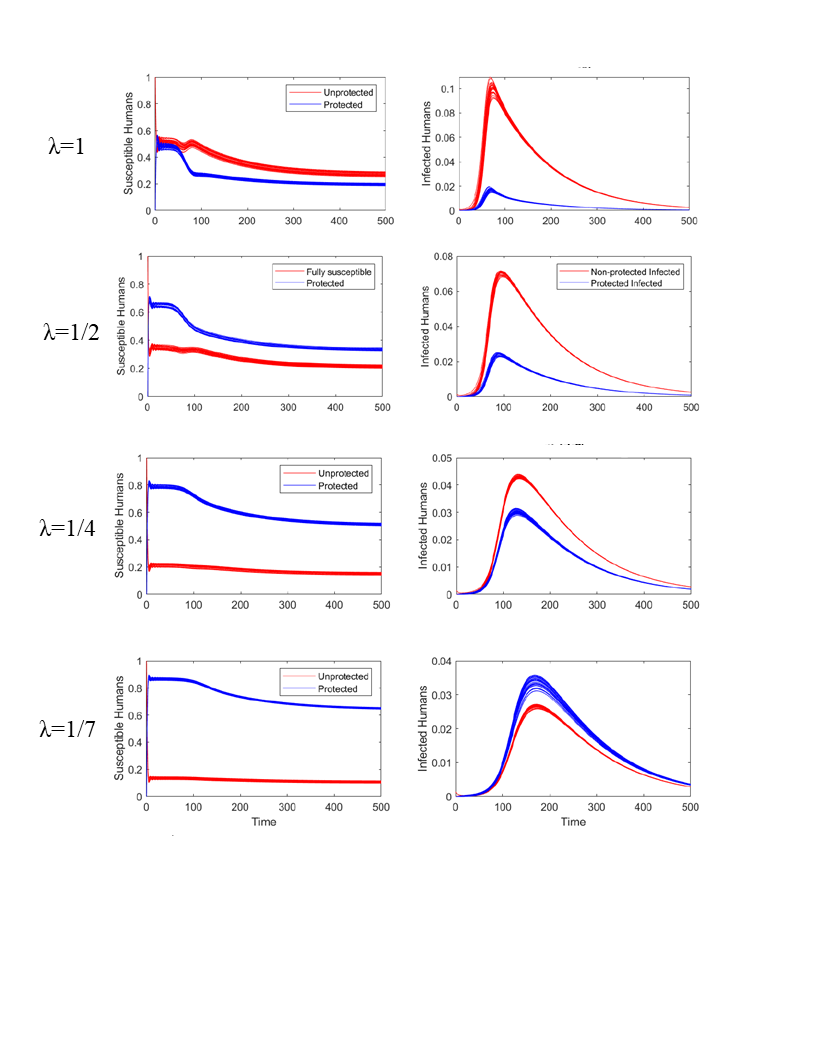


**Figure S2.** Dynamics of the susceptible and infectious populations for increasing values of p, or the probability of mobility, keeping all other parameter values the same, for scenarios with high community control, and medium concerns for biting and disease transmission. Blue curves represent protected individuals, while red represents unprotected.


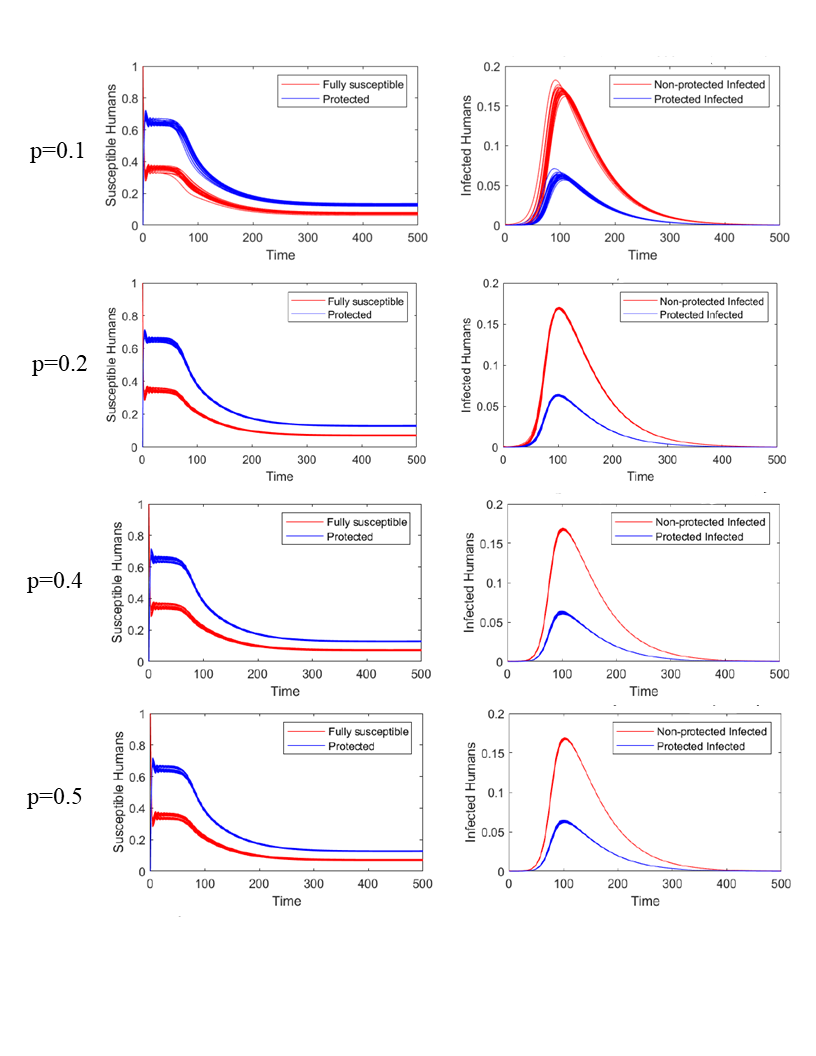


**Figure S3.** Human and vector population dynamics representing low levels of community-level control. This is also for the scenario with low concern for both drivers of personal protection use, to primarily show the dynamics of community-level control.

**
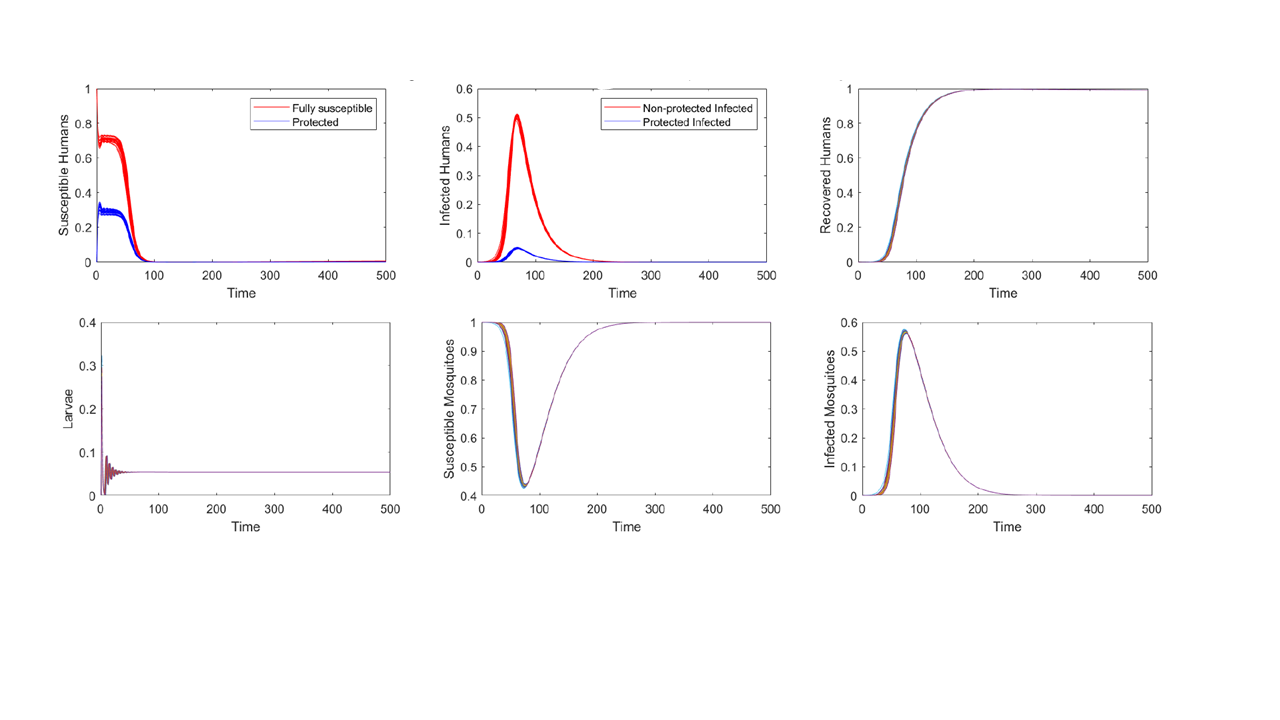
**

**Figure S4.** Human and vector population dynamics representing medium levels of community-level control. This is also for the scenario with low concern for both drivers of personal protection use, to primarily show the dynamics of community-level control.

**
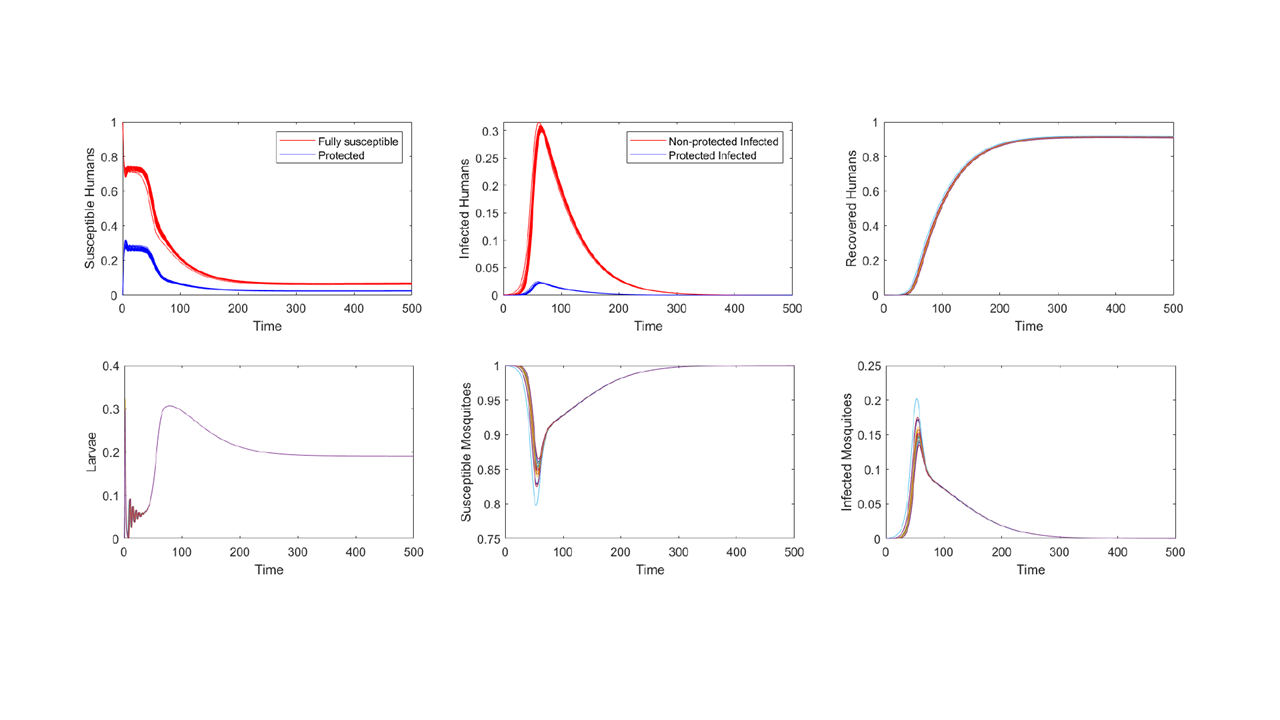
**

**Figure S5.** Human and vector population dynamics representing high levels of community-level control. This is also for the scenario with low concern for both drivers of personal protection use, to primarily show the dynamics of community-level control.

**
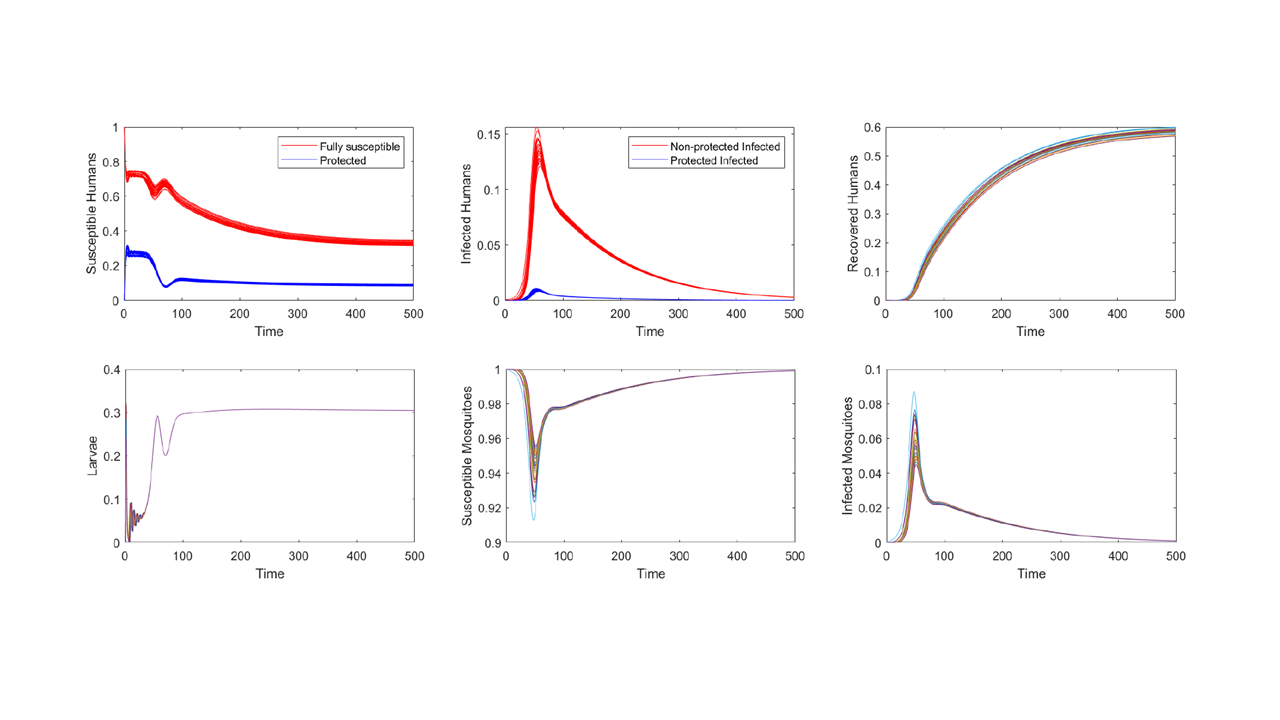
**

***References***

1. Suarez GP, Udiani O, Allan BF, Price C, Ryan SJ, Lofgren E, et al. A generic arboviral model framework for exploring trade-offs between vector control and environmental concerns. J Theor Biol. 2020;490:110161.
